# Abnormal Origin of the Right Pulmonary Artery From Ascending Aorta (Hemitruncus Arteriosus)

**DOI:** 10.1177/2324709614536139

**Published:** 2014-07-27

**Authors:** L. Julian Haywood, Yervand Chakryan, Diana Kim, Tin Boltzer, Gregory Rivas, David Shavelle

**Affiliations:** 1 Keck School of Medicine, University of Southern California, Los Angeles, CA, USA; 2 LAC+USC Medical Center, Los Angeles, CA, USA

**Keywords:** hemitruncus arteriosus, anomalous pulmonary artery, congenital heart disease, patent ductus arteriosis, pulmonary hypertension

## Abstract

Hemitruncus arteriosus is a rare congenital deformity that results in early infant mortality. Persistence into adulthood is very unusual and is associated with pulmonary hypertension. We report a case in an adult male with the associated clinical issues.

## Introduction

Hemitruncus arteriosus refers to pulmonary artery originating from aorta and is a rare congenital cardiovascular malformation that presents in infancy. This anomaly was first described by Fraentzel in 1868,^1^ and more than 95% of the reported cases of hemitruncus arteriosus diagnosed are at infancy. There have only been a few case reports of adults with natural progression of this anomaly.

Right pulmonary artery anomaly is more common and it occurs when it abnormally arises from the posterior aspect of the ascending aorta very near the aortic valve. Less commonly, the left pulmonary artery anomaly originates from the ascending aorta and is usually associated with a right aortic arch.^2^ The lung that is supplied from right ventricle carries full systemic volume and the lung supplied by the anomalous originating pulmonary artery delivers systemic blood pressures. The natural history of an anomalous origin of one pulmonary artery from aorta includes development of progressive pulmonary vascular disease and heart failure and death. Infants who do not undergo surgical correction have a 70% first-year mortality rate, and 30% of the infants die within 3 months.^3^ Infants diagnosed with this anomaly undergo early surgical repair, which leads to rapid physiologic correction.^3-5^

## Case Description

A 33-year-old Mexican male with history of “murmur” as a child, occasional hemoptysis, worsening dyspnea with exertion and at high altitude, presented with dyspnea and oxygen saturation of 88%. The patient described increased episodes of hemoptysis over the past 2 years; however, he had not previously sought medical attention given the self-resolving nature of these episodes. The patient initially presented to a community clinic and there a bedside transthoracic echocardiogram reported right heart hypertrophy and pulmonary stenosis with possible right ventricular thrombus. He was subsequently referred to our LAC+USC Medical Center. At the time of arrival, the patient did not appear in acute distress. His physical examination revealed no cardiac murmur, no jugular venous distention, positive thrill at left sternal border, and minimal clubbing without cyanosis of bilateral toes.

**Figure 1.**
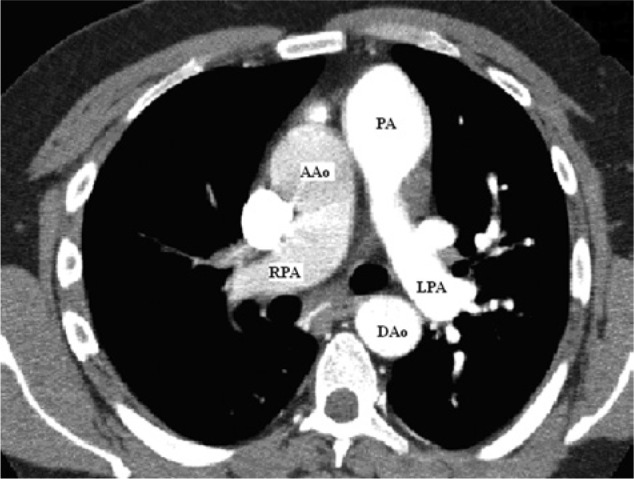
Right pulmonary artery (RPA), left pulmonary artery (LPA), pulmonary artery (main; PA), ascending aorta (AAo), and descending aorta (DAo).

**Figure 2.**
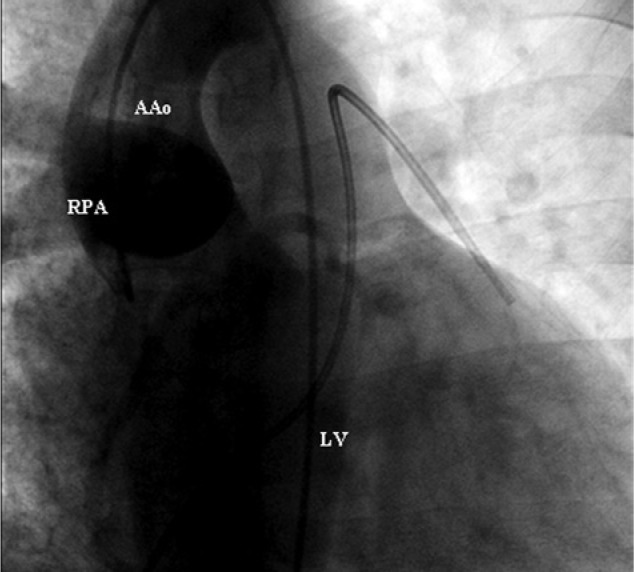
Right pulmonary artery (RPA), ascending aorta (AAo), and left ventricle (LV).

**Figure 3.**
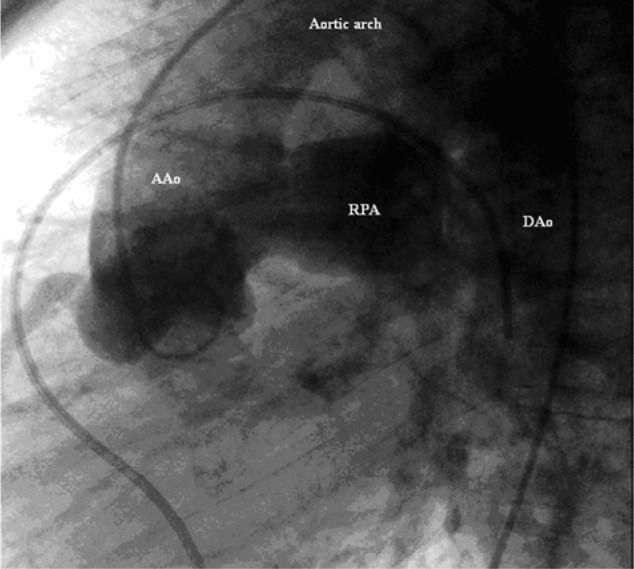
Cardiac catheterization showing anomalous origin of right pulmonary artery (RPA); ascending aorta (AAo), descending aorta (DAo).

Repeat transthoracic echocardiogram revealed severe right ventricular hypertrophy with increased right ventricular pressure. There was no evidence of valvular abnormalities or ventricular thrombus, and systolic function was preserved. The patient later underwent a computed tomography pulmonary angiogram, which was negative for pulmonary embolism; however, it did revealed a tubular patent ductus arteriosus (PDA) and an anomalous right pulmonary artery stemming from the ascending aorta (Figure 1). Later, cardiac catheterization confirmed hemitruncus anomaly (Figures 2 and 3) and large PDA with primarily right to left flow. Furthermore, the catheterization revealed severe pulmonary hypertension; right ventricular systolic pressure of 150 mm Hg, left pulmonary artery pressure 145 mm Hg, and right pulmonary artery pressure of 100 mm Hg.

Cardiac catheterization also demonstrated severely diminished blood oxygen saturation: left pulmonary artery 39%, left ventricle 80%, aortic arch 78%, and femoral artery 50%. This indicated that the elevated pulmonary pressures had disrupted lung parenchyma thus creating shunting of blood without normal oxygenation. The case was discussed in cardiology/cardiothoracic conference for possible surgical intervention.

## Discussion

Hemitructus arteriosus is a very rare congenital cardiovascular malformation and almost all the documented cases have been in infants. In the literature survey we conducted, we found only 10 cases of hemitruncus arteriosus in adults.^6-9^ Presentation in adulthood is usually recurrent hemoptysis and dyspnea,^6^ which was the primary complaint of our patient.

**Figure 4.**
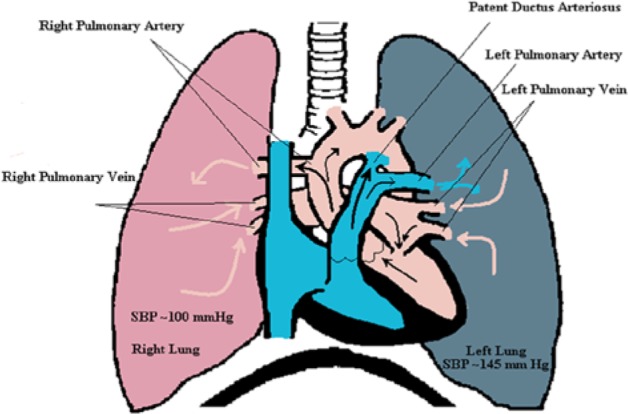
Graphic illustration of the patient’s anomaly.

In our patient, the anomaly created 2 separate blood circuits to each lung (Figure 4). The left lung received all of systemic blood volume from the right ventricle and the right lung received oxygenated blood from the left ventricle, which circulated back to left atrium. This lifelong systemic volume flow to the left lung caused progressive worsening of pulmonary hypertension and right ventricular hypertrophy. In contrast, the right lung received blood flow through the anomalous right pulmonary artery; however, as a result of systemic pressure from the left ventricle, it too developed pulmonary hypertension.

Our case was discussed in cardiology conference and with expert opinion we concluded that the prolonged left lung hypertension likely caused the lung parenchymal disease and shunting, thus progressing to poor capability to oxygenate blood. It is not likely that the right lung contributed to significant blood oxygenation given the finding of 80% blood oxygenation in the left ventricle shown by the cardiac catheterization. In addition, the PDA complicated the picture, since the right ventricular hypertrophy caused an Eisenmerger physiology, and right to left shunting further complicated deoxygenation as seen by the 78% blood oxygenation in the aortic arch.

On further discussion with cardiothoracic surgeons, it was suggested that the patient may benefit from undergoing double lung transplant with the correction of right pulmonary artery origin and the ligation of the PDA. This would eliminate the pulmonary hypertension and problem with blood oxygenation, which would then lead to the remodeling of right heart myocardium.

In one reported case of a 41-year-old patient with recurrent hemoptysis originating from the right lung, secondary to increased pulmonary hypertension from the anomalous right pulmonary arteru, palliative banding of right pulmonary artery was undertaken. Right pulmonary artery banding reduced the right pulmonary pressure and a provided symptomatic relief. It was concluded that surgical intervention had high mortality/morbidity risk and palliative procedure would be more appropriate in an adult with hemitruncus. Per reports, the procedure was a success and the patient had not experienced any further hemoptysis since surgery.^6^ However, the difference between our patient and the one reported by Nikolaidis et al^6^ was that the latter did not have right ventricular hypertrophy, pulmonary hypertension in the left “normal” lung, or poor blood oxygenation.

Our patient remained under observation, was hospitalized for 1 week, and was asymptomatic without complaints of dyspnea or episodes of hemoptysis. The patient’s blood oxygenation was 88% to 90% on room air and he was discharged home with follow-up. Further discussion will take place with patient and cardiothoracic surgical team in pursuit of surgical intervention.

Our patient did not agree to be considered for the surgical correction recommended stating that he felt well most of the time, and did not keep his follow-up appointment. Contacted recently, the patient stated that he has been followed by a cardiologist closer to his home and that his symptoms have recently increased; he has accepted an appointment to return to our medical center for further evaluation.

## References

[1] Fraentzel O Ein Fall Von Abnormer communication Der Aorta mit der Arteria Pulmonalis. Arch Pathol Anat Physiol Klin Med. 1868;43:420-426.

[2] Prifti E Bonacchi M Murzi B Anomalous origin of the right pulmonary artery from the ascending aorta. J Card Surg. 2004;19:103-112. 15016045 10.1111/j.0886-0440.2004.04023.x

[3] Fontana GP Spach MS Effmann EL Sabiston DC Origin of the right pulmonary artery from the ascending aorta. Ann Surg. 1987;206:102-113. 3606229 10.1097/00000658-198707000-00016 PMC1492932

[4] Amir G Frenkel G Bruckheimer E Anomalous origin of the pulmonary artery from the aorta: early diagnosis and repair leading to immediate physiological correction. Cardiol Young. 2010;20:1-6. 10.1017/S1047951110000892 20723270

[5] Peng EW Shanmugam G Macarthur KJ Pollock JC Ascending aortic origin of a branch pulmonary artery—surgical management and long term outcome. Eur J Cardiothorac Surg. 2004;26:762-766. 15450569 10.1016/j.ejcts.2004.07.007

[6] Nikolaidis N Velissaris T Haw MP Pulmonary artery banding for hemi-truncus arteriosus in adulthood. Thorac Cardiovasc Surg. 2010;58:181-183. 20376732 10.1055/s-0029-1240746

[7] Edasery B Sharma M Vaddigiri V Santucci T Jr Hemitruncus in an adult. Angiology. 1996;47:1023-1026. 8873589 10.1177/000331979604701012

[8] Wu M Yang G Origin of the right pulmonary artery from the ascending aorta in a 25-year-old man. Texas Heart Inst J. 2006;33:534-535. PMC1764951 17215991

[9] Sectem U Jungehulsing M De Vivie R Mennickens U Hopp HW Left hemitruncus in adulthood: diagnostic role of magnetic resonance imaging. Eur Heart J. 1991;12:1040-1044. 1936004

